# Stomatal CO_2_ sensing in plants: control of gas exchange and interactions with environmental stimuli

**DOI:** 10.1093/pcp/pcaf074

**Published:** 2025-07-03

**Authors:** Yohei Takahashi, Hyunhee Joo, Nattiwong Pankasem, Po-Kai Hsu, Julian I Schroeder

**Affiliations:** Institute of Transformative Bio-Molecules (WPI-ITbM), Nagoya University, Chikusa, Nagoya 464-8601, Japan; Division of Biological Science, Graduate School of Science, Nagoya University, Chikusa, Nagoya 464-8602, Japan; Cell and Developmental Biology Department, School of Biological Sciences, University of California San Diego, 9500 Gilman Drive, La Jolla, CA 92093-0116, USA; Cell and Developmental Biology Department, School of Biological Sciences, University of California San Diego, 9500 Gilman Drive, La Jolla, CA 92093-0116, USA; Cell and Developmental Biology Department, School of Biological Sciences, University of California San Diego, 9500 Gilman Drive, La Jolla, CA 92093-0116, USA; Cell and Developmental Biology Department, School of Biological Sciences, University of California San Diego, 9500 Gilman Drive, La Jolla, CA 92093-0116, USA

**Keywords:** carbon dioxide (CO_2_), CO_2_/HCO_3_^−^ sensor, guard cells, protein kinase, stomata, water use

## Abstract

Stomatal pores in land plants rapidly and reversibly open and close in response to diurnal changes in leaf carbon dioxide (CO_2_) concentration. Studies have suggested that CO_2_ is sensed by guard cells with relevant amplifying contributions from mesophyll tissue. CO_2_ concentration changes trigger rapid signal transduction events involving protein phosphorylation in guard cells. Moreover, molecular crosstalk and physiological interactions of the stomatal CO_2_ response with other environmental conditions and stimuli, including light, temperature, drought, and abscisic acid, are reviewed here. Genetic studies have revealed several key genes and provided important insights into the stomatal CO_2_ sensors and signal transduction mechanisms. The primary CO_2_/HCO_3_^−^ sensor in Arabidopsis guard cells was recently identified. Quantitative trait locus (QTL) analyses have shown that early guard cell CO_2_ signal transduction components regulate water use efficiency (WUE). In this review, we describe the molecular details of stomatal CO_2_ sensing by CO_2_/HCO_3_^−^-induced interaction of two protein kinases, the HIGH LEAF TEMPERATURE 1 Raf-like kinase and the MPK4/MPK12 mitogen-activated protein kinases. The evolutionary emergence of, physiological relevance of, and potential for improvement of WUE of plants via the stomatal CO_2_ response and open questions in this research field are discussed.

## Introduction

Stomatal pores control the flow of gases and water vapor between plants and the environment. Each stomatal pore is encircled by two guard cells on aerial epidermal surfaces of nearly all land plants. The fossil record indicates that stomata are both ancient and are a significantly conserved feature of terrestrial plants (Beerling and Royer 2002). As plants transitioned from aquatic to terrestrial habitats, they encountered a range of environmental challenges, including desiccation (Waters 2003). In response, plants evolved a hydrophobic layer on the surface of the epidermis known as the cuticle to reduce water loss (Kong et al. 2020). Such cuticular surfaces limit carbon dioxide (CO_2_) entry and water vapor loss, which led to the necessity for stomata in land plants (Riederer and Muller 2008, Clark et al. 2022).

Stomata respond quickly to changes in CO_2_ concentrations in leaf intercellular air spaces. High CO_2_ triggers stomatal closing, and low CO_2_ induces stomatal opening (Heath 1948). The CO_2_ concentration inside leaves (*c_i_*) shifts rapidly on a daily basis, e.g. upon cloud cover or darkness when respiration and reduced photosynthesis quickly cause a doubling or more in *c_i_* (Hanstein et al. 2001, Roelfsema et al. 2002). Since the beginning of the Industrial Revolution, atmospheric CO_2_ concentrations have shown a steep rise. Global atmospheric CO_2_ has increased from 315 ppm to more than 420 ppm since 1958, according to recordings at the Mauna Loa Volcanic Observatory. The CO_2_ concentration is predicted to be 800 ppm or higher by the end of this century. This increase in ambient CO_2_ concentrations is expected to reduce stomatal pore apertures and stomatal density in many plant species (Medlyn et al. 2001, Ainsworth and Rogers 2007). Stomata play physiologically important roles in various aspects of plant life, including the exchange of CO_2_ and water vapor and facilitating transpiration, which can facilitate the uptake of water and nutrients from the soil (Hetherington and Woodward 2003). Furthermore, stomatal transpiration reduces leaf temperatures. Therefore, understanding stomatal responses to CO_2_ concentrations has been one of the critical research areas concerning the impacts of frequently occurring weather extremes on plant growth and crop yield.

As autotrophic organisms, plants survive and grow due to photosynthesis. The biochemical pathways of photosynthesis to harness light energy for the conversion of CO₂ into sugars have been preserved through evolutionary transitions from cyanobacteria to chloroplasts in higher plants (Vermaas 2001). Yet photosynthesis has undergone significant evolutionary changes over an extended period of time, resulting in highly intricate and sophisticated mechanisms. Given that CO_2_ is required in the process of carbon assimilation, the regulatory mechanisms that control stomata, i.e. the small openings on the surface of plants through which gases exchange between the atmosphere and the leaves, are inextricably linked to the process of photosynthesis.

In this review, we discuss the evolutionary and physiological aspects of stomatal movements in response to changes in CO_2_ concentration and review the advances in research on stomatal CO_2_ responses. Given the recent discovery of the stomatal primary CO_2_/HCO_3_^−^ sensor in Arabidopsis, we describe the molecular mechanisms of how changes in CO_2_ concentration can be perceived by stomatal guard cells and induce stomatal movements.

## Evolutionary Aspects of Stomatal Function

### Stomata in non-vascular and vascular land plants

After the appearance of plants with stomata, plant lineages diverged into non-vascular plants such as bryophytes and vascular plants with roots and vascular tissue (Berry et al. 2010, Clark et al. 2022). As a non-vascular land plant, the moss (*Physcomitrium patens)* belongs to one of the most primordial diverging lineages that possess stomata (Caine et al. 2016). In *P. patens*, stomata are localized to the capsule on the sporophyte (Chater et al. 2016). It has been known that moss stomata play a critical role in the desiccation of the capsule, which leads to the dispersal of spores (Duckett et al. 2009, Chater et al. 2016). According to recent studies, the moss *P. patens* shares a common ancestral molecular machinery for stomatal development with Arabidopsis (Caine et al. 2016, Chater et al. 2016). Specifically, the presence of orthologs *PpSMF1* and *PpSCRM1* are necessary for moss stomata development, paralleling Arabidopsis regulatory processes (Pillitteri and Torii 2012, Chater et al. 2016, Simmons and Bergmann 2016). More recent studies have shown that moss stomata regulate CO_2_ assimilation and water loss (Kubásek et al. 2021). Regarding the evolution of stomatal opening and closing, there are conflicting views on stomatal function. The phytohormone abscisic acid (ABA) induces stomatal closure in response to drought (Cutler et al. 2010). Brodribb and McAdam (2011) argued that bryophyte, lycophyte, and fern stomata lack ABA and elevated CO_2_ responses. They proposed that stomatal responses in these groups occur solely via “hydropassive” mechanisms and that receptor/sensor-mediated “hydroactive” signaling mechanisms emerged only with gymnosperms and angiosperms (Brodribb and McAdam 2011, McAdam and Brodribb 2012). Conversely, other studies suggest that early-developing lineages utilized additional hydroactive mechanisms for stomatal responses—opening and closing in response to ABA, CO_2_, and light, suggesting their presence in early land plants (Chater et al. 2011, Ruszala et al. 2011, Clark et al. 2022). Research on stomatal responses in intact fern plants has shown unequivocal CO_2_ and ABA responses in some fern species depending on conditions, but a lack of or weak responses in other fern species (Horak et al. 2017). In seed plants, the presence of active regulators of stomatal responses has been substantiated by various findings (Tardieu and Davies 1993, Negi et al. 2008, Vahisalu et al. 2008).

### History of atmospheric CO_2_ concentration and the emergence of stomata

It has been suggested that a major reduction in atmospheric CO_2_ concentration occurred 400 million years ago during the mid-Paleozoic era (Beerling 2005). A substantial reduction in CO_2_ levels during the mid-Paleozoic was due to increased silicate weathering, with secondary contributions from enhanced sequestration of organic matter resulting from photosynthesis via the proliferation and dispersal of vascular terrestrial plants (Berner 1991, Berner 1992). The emergence of stomata ~400 million years ago enabled plants to increase CO_2_ diffusion linked to the assimilation of CO_2_ necessary for photosynthesis, while simultaneously managing water loss through transpiration (Hetherington and Woodward 2003). This pivotal evolutionary advancement significantly aided the establishment of vascular plants in terrestrial ecosystems (Edwards et al. 1998, Duckett et al. 2009, Chater et al. 2016).

### Evolution of CO_2_ signaling components

Recent studies have identified MPK4/12 and HIGH LEAF TEMPERATURE 1 (HT1) as primary sensors in CO_2_ signaling pathways, with CONVERGENCE OF BLUE LIGHT AND CO_2_ 1/2 (CBC1/2) acting downstream of HT1 to mediate stomatal responses to CO_2_ (see Sections “Identification of key signaling protein kinases” and “HT1-MPK4/12 complex is the primary CO_2_/HCO_3_^−^ sensor” for details; Takahashi et al. 2022). The *MAPK* gene family is conserved across all major plant lineages. However, *MPK4* and *MPK12* orthologs, which function in stomatal regulation, appear to have evolved more recently within angiosperms (González-Coronel et al. 2021, Ivsic et al. 2025). The Raf-like *MAPKKK* genes, *HT1* and *CBC1/2*, have undergone distinct evolutionary processes. Phylogenetic analyses have shown that *CBC1/2* are found in both gymnosperms and angiosperms, while CO_2_-sensing *HT1* is found only in angiosperms (Ivsic et al. 2025). Given that non-angiosperms show a relatively weak (or absent) stomatal CO_2_ response (Klein and Ramon 2019), a comparative phylogenetic analysis of the stomatal CO_2_/HCO_3_^−^ sensing HT1-MPK12/4 module along with downstream signaling components across plant species would provide additional insights on CO_2_ sensing and signaling mechanisms in non-angiosperm plants.

## Stomatal CO_2_ Signaling Mechanisms

### Rapid and reversible stomatal movements in response to CO_2_

**Figure 1:**
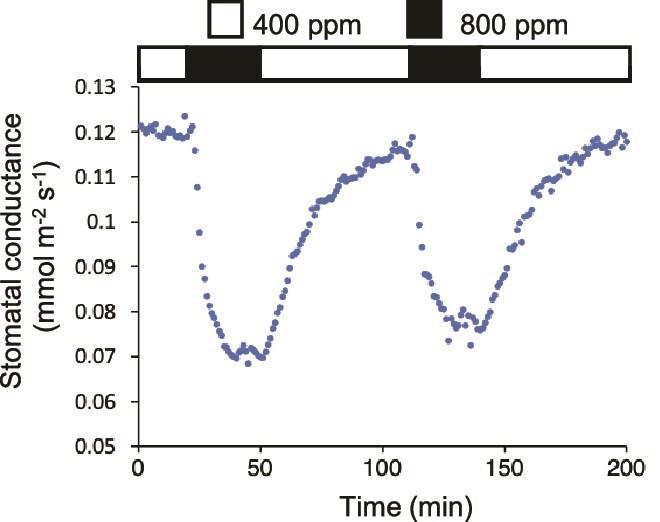
Reversible stomatal response to changes in CO_2_ concentration. Effect of ambient CO_2_ concentration shifts on stomatal conductance of an Arabidopsis leaf was measured using a gas exchange analyzer (LI-6800, LI-COR, Nebraska, USA). The stomatal conductance was stabilized under 400 ppm CO_2_. The external CO_2_ concentration was changed to 800 ppm and then returned to 400 ppm repeatedly as indicated above. Stomatal conductance data were collected every minute.

Similar to the stomatal response to light and dark, the opening and closing of stomata in response to changes in CO_2_ concentration are rapid and reversible (Fig. 1). In general, stomata open when the CO_2_ concentration decreases and close in response to CO_2_ elevation. Although it is unlikely that atmospheric CO_2_ concentrations drastically fluctuate throughout a single plant’s life (Mott 1990), the CO_2_ concentration in plant leaves can dynamically change due to variations in photosynthetic activity and respiration depending on light conditions (Mott 1990, Hanstein et al. 2001, Roelfsema et al. 2002). Stomatal guard cells can sense these changes in CO_2_ concentration within the leaf. The rapid and reversible stomatal movements allow plants to control stomatal apertures in response to carbon demand for photosynthesis, which is advantageous in balancing the uptake of CO_2_ for photosynthesis and water loss through the stomatal pores. The stomatal closure rate in Arabidopsis in response to high CO_2_ concentrations is usually faster than the low CO_2_-induced stomatal opening rate (Fig. 1). Stomatal closure is achieved by rapid passive ion transport through ion channels, resulting in water efflux and a decrease in guard cell volume, whereas stomatal opening requires slower active proton pump-energized ion transport against concentration gradients into the cells and an increase in guard cell volume (Shimazaki et al. 2007). The signal transduction mechanisms underpinning stomatal opening and closing in response to CO_2_ concentration must be rapid and reversible.

### Identification of key signaling protein kinases

The CO_2_ sensing and signal transduction mechanisms had long remained unclear. However, a high-throughput unbiased forward genetic screening system for mutants with impaired stomatal function was established in 2002 by measuring leaf surface temperature using infrared thermography (Merlot et al. 2002), and a pioneering study applied this methodology to stomatal CO_2_ response research (Hashimoto et al. 2006). Hashimoto et al. (2006) used thermography to isolate recessive Arabidopsis mutants whose stomata did not open at low CO_2_ concentrations and isolated a gene named *HT1* (Hashimoto et al. 2006). The identification of the *HT1* gene has greatly advanced plant CO_2_ research, and the function of HT1 as a direct component of the primary stomatal CO_2_ sensor was recently revealed, as discussed below.

The *HT1* gene was identified as one of the protein kinase genes belonging to the subgroup C5 of the Arabidopsis Raf-like gene family (MAPK Group 2002, Hashimoto et al. 2006). Strong recessive *ht1* mutant alleles show a constitutive high CO_2_ closed stomatal response and disrupt low CO_2_-induced stomatal opening (Hashimoto et al. 2006). Subsequently, genetic screening led to the identification of dominant *HT1* point mutations (*R102K* and *A109V*) that caused the opposite phenotype to recessive *ht1* mutant alleles (Hashimoto-Sugimoto et al. 2016, Hõrak et al. 2016). These dominant *HT1* alleles caused a constitutive stomatal opening and disruption of high CO_2_-induced stomatal closing (Hashimoto-Sugimoto et al. 2016, Hõrak et al. 2016), in contrast to the constitutively closed stomata of the *ht1* loss-of-function plants. The findings that the dominant point mutations in *HT1* do not greatly affect HT1 kinase activity *in vitro* (Hashimoto-Sugimoto et al. 2016, Hõrak et al. 2016, Takahashi et al. 2022) suggest that proper regulation of HT1 kinase activity in response to CO_2_ might be required for the stomatal CO_2_ response.

Arabidopsis CBC1/2 kinases, which belong to the subgroup C7 of the Raf-like kinase family (MAPK Group 2002, Hiyama et al. 2017), have a similar CO_2_ signaling phenotype to that of the HT1 protein kinase. CBC1 and CBC2 were identified by a phosphoproteomics approach. The *cbc1 cbc2* double knockout mutant has constitutively closed stomata regardless of CO_2_ concentration, similar to the *ht1* loss-of-function mutants (Hiyama et al. 2017). CBC1 is phosphorylated in response to blue light (Hiyama et al. 2017). Although the physiological significance of the blue-light-induced phosphorylation of CBC1 remains unclear, CBC1 can function as a convergence point between stomatal blue light and CO_2_ signaling pathways (Hiyama et al. 2017).

The mitogen-activated protein kinases, MPK4 and MPK12, belong to the subgroup B of the 20 MAP kinase genes in Arabidopsis (MAPK Group 2002), have a phenotypically opposite function to the HT1 kinase and CBC1/2 kinases. The *mpk12-4* single gene loss-of-function mutant showed greater stomatal conductance and weak CO_2_ sensitivity (Jakobson et al. 2016), and guard-cell-specific knockdown of the clade member, *MPK4*, in the *mpk12-4* mutant caused a high stomatal conductance and largely disruption of CO_2_-induced stomatal closing (Tõldsepp et al. 2018). Interestingly, *mpk12* was also found as a background mutation in some GABI-KAT lines. The phenotypic discrepancies among different *calcium-sensing receptor* (*cas*) alleles (Jakobson et al. 2016) and between different *glutamate decarboxylase 2* (*gad2*) alleles (Piechatzek et al. 2024) in stomatal CO_2_ response led to the identification of the lack of *MPK12* as the genuine cause of their CO_2_-insensitive phenotypes. *mpk12* loss-of-function mutants and natural variants showed reduced water use efficiency (WUE) (Des Marais et al. 2014, Jakobson et al. 2016), indicating the physiological importance of the stomatal CO_2_ response in balancing water loss and carbon uptake. It was also reported that silencing the *MPK4* ortholog in *Nicotiana tabacum* resulted in stomatal opening and CO_2_ insensitivity (Marten et al. 2008). Interestingly, the stomata of *mpk4/12* showed robust stomatal closure in response to ABA (Tõldsepp et al. 2018).

### Second messengers

Possible second messengers and their functions in the stomatal CO_2_ response have been discussed intensely in other papers (Assmann 1999, Vavasseur and Raghavendra 2005). We review this subject only briefly below:

#### Cytosolic calcium ion concentration

Calcium is a universal second messenger in signal transduction. Several studies showed that elevation in the cytosolic calcium concentration contributes to stomatal closure. In 1996, Webb et al. reported that intracellular Ca^2+^ increased in response to high CO_2_ concentrations in stomatal guard cells of *Commelina communis* using fura-2 fluorescence imaging (Webb et al. 1996). Calcium can mediate stomatal closure by regulating ion channels in guard cells (Schroeder and Hagiwara 1989). Intriguingly, modulation of calcium transients was observed during low and high CO_2_ concentrations that lead to stomatal opening and closing in Arabidopsis guard cells (Young et al. 2006). It is still a mystery how calcium signaling can trigger a specific physiological response. One possible hypothesis is stimulus-specific regulation (priming) of Ca^2+^-sensitive proteins (Young et al. 2006, Dodd et al. 2010). Quintuple mutants in calcium-dependent protein kinases showed a slowed stomatal CO_2_ response, whereas knockout of all five plasma membrane (PM)-targeted Ca^2+^-binding CBL proteins did not impair the stomatal CO_2_ response (Schulze et al. 2021).

#### Cytosolic pH change

CO_2_ lowers the pH in the solution when it dissolves. Since ion transporters in stomatal guard cells can be controlled by pH changes, cytosolic pH has been investigated as a possible second messenger in stomatal CO_2_ responses. However, it remains unclear whether a decrease in pH mediates stomatal CO_2_ signaling (Assmann 1999). The change in cytoplasmic pH caused by physiological concentrations of CO_2_ seems to be almost negligible in guard cells (Brearley et al. 1997, Xue et al. 2011).

#### Malate

When the CO_2_ concentration rises, the apoplastic carbon metabolite malate was reported to increase from about 1 mM up to about 3 mM in *Vicia faba* (Hedrich et al. 1994). Extracellular malate has been reported to activate the PM anion channels in guard cells, leading to anion efflux from guard cells and resulting in stomatal closure (Hedrich and Marten 1993). These findings led to a hypothesis that malate might be the CO_2_ sensor that mediates stomatal movements in response to changes in CO_2_ concentration (Hedrich and Marten 1993). However, other research reported that very high concentrations (40 mM or more) of apoplastic malate were needed for stomatal closure (Cousson 2000). Therefore, whether the increase in apoplastic malate explains stomatal CO_2_ sensing has been unclear.

ABC transporters mediate export of molecules from the cytoplasm. In contrast, the PM-localized transporter ABCB14 was proposed to mediate influx of malate into guard cells (Lee et al. 2008). Loss-of-function mutants of the PM-localized transporter ABCB14 were reported to show a slightly lower stomatal conductance at a high CO_2_ concentration than wild-type control plants (Lee et al. 2008). However, when isolated epidermis was exposed to high concentrations of CO_2_, CO_2_-induced stomatal closure similar to that of the wild type was observed. On the other hand, when the isolated epidermis was exposed to malate, the knockout plants closed stomata more tightly than the wild type. Malate produced in mesophyll cells may be involved in malate-dependent stomatal closure through the apoplastic pathway (Lee et al. 2008), but as discussed above, physiological concentrations of extracellular malate alone did have a consistent effect on CO_2_-regulated stomatal movements (Cousson 2000). Evidence from several laboratories suggests that crosstalk between guard cells and mesophyll cells is involved in CO_2_ control of stomatal movements, with sugar supply to guard cells from the mesophyll playing an important role (Messinger et al. 2006, Flutsch et al. 2020a, 2020b).

### CO_2_ regulation of the PM potential in guard cells

Electrophysiological analysis has shown that the membrane potential of the guard cell PM becomes more positive (depolarizes) in response to high CO_2_ (Brearley et al. 1997, Roelfsema et al. 2002). Consistent with these findings, electrophysiological analyses have shown that both S-type and R-type anion channels in guard cells are activated by CO_2_ elevation (Raschke et al. 2003). Anion channel-mediated anion efflux causes depolarization of the guard cell PM.

It was suggested that the PM H^+^-ATPase is controlled by CO_2_ (Edwards and Bowling 1985). Although this is likely, direct regulation of PM H^+^-ATPases during CO_2_ signaling has not yet been reported, likely due to the difficulties in measuring this activity in the presence of other conductances and in biochemically investigating its CO_2_ response in guard cells. Recent immunostaining has reported that the penultimate threonine residue of the PM proton pump of *Arabidopsis thaliana* can be dephosphorylated in response to the addition of NaHCO_3_ (Ando et al. 2022). However, studies suggest that HT1 and CBC1/2 protein kinases do not function in phosphorylation of the penultimate threonine residue of the PM H^+^-ATPase (Hiyama et al. 2017, Ando et al. 2022). Further research is required to link early CO_2_ signal transduction mechanisms to proton pump regulation.

## What Is the Primary Stomatal CO_2_ Sensor?

### CO_2_ sensing in other organisms

Many organisms, including humans, sense CO_2_/bicarbonate for homeostasis and environmental responses. Soluble adenylyl cyclase (sAC) is an enzyme responsible for producing cyclic adenosine monophosphate (cAMP), one of the common second messengers of intracellular signal transduction across species. sAC was found to be activated by bicarbonate in mammals (Chen et al. 2000) and functions as a CO_2_/HCO_3_^−^ sensor (Rossetti et al. 2021). The crystal structure of the human sAC with bicarbonate ions was solved in 2014 (Kleinboelting et al. 2014). In insects such as mosquitoes and fruit flies, gustatory receptors sense CO_2_ (Jones et al. 2007). However, no homologous proteins with similar functions have been found in higher plants, suggesting independent evolutions of CO_2_ sensing components in plants and animals.

### Rubisco

Most guard cells have chloroplasts with metabolic functions but likely with reduced photosynthetic activity compared to mesophyll cells (Reckmann et al. 1990, Tominaga et al. 2001, Lawson 2009). It has been demonstrated that photosynthesis in guard cells is required for maintaining stomatal turgor pressure rather than for CO_2_ signaling (Azoulay-Shemer et al. 2015). A role for the weaker photosynthetic activity of guard cells in stomatal opening is supported by the finding that chlorophyll-depleted guard cells can show collapsed closed stomatal apertures (Azoulay-Shemer et al. 2015). Rubisco is an enzyme that captures CO_2_ as the first step of the Calvin cycle in C3 plants. Therefore, Rubisco could theoretically function as a guard cell CO_2_ sensor candidate. Mutants in guard cell-expressed Rubisco small subunit genes did not show a clear impairment in high CO_2_-induced stomatal closing (Von Caemmerer et al. 2004, Hu et al. 2010). These findings suggest that the CO_2_ sensor mechanism that controls stomatal closing is mediated by a different pathway.

### Carbonic anhydrases and bicarbonate

Mechanisms that detect variations in CO_2_ and bicarbonate concentrations regulate stomatal movements. Carbonic anhydrases (CAs) are enzymes that facilitate the reversible conversion of CO_2_ and water into protons and bicarbonate ions. Double mutants in the beta-carbonic anhydrases βCA1 and βCA4 show a higher stomatal conductance and slowed CO_2_-induced stomatal closure in Arabidopsis (Hu et al. 2010). Notably, introducing a structurally unrelated mammalian CA into double-mutant plants by expression in guard cells effectively rescued the wild-type-like stomatal CO_2_ response (Hu et al. 2010). This evidence suggests that the catalytic function of CA enzymes plays a critical role in “transponding” (Frommer 2010) the CO_2_ signal within this pathway and implicates bicarbonate and/or protons as potential second messengers in the process of signal transduction. Hu et al. (2015) investigated the intracellular localization of beta-carbonic anhydrases βCA1 and βCA4 in guard cells, revealing that distinct cellular expression localizations can restore CO_2_-induced stomatal responses. Guard cell localization analyses and reaction–diffusion modeling led to the model that more than one cellular localization, including PM or cytoplasmic localization of CAs, enables CO_2_ control of stomatal movements (Hu et al. 2015). A double mutant in maize beta-CA (βCA) showed impaired stomatal CO_2_ responses (Kolbe et al. 2018). These maize beta-CAs are predicted to be located in the cytoplasm, consistent with this model. Moreover, mutation of the rice carbonic anhydrase βCA1 showed impairment in CO_2_-regulated stomatal movements consistent with these studies (Chen et al. 2017). Interestingly, different splice isoforms of rice βCA1 are targeted to different locations in guard cells that rescue the stomatal CO_2_ response (Mao et al. 2025). Given that β-CAs use their enzymatic activity to accelerate the catalysis of CO_2_, this research suggested that the stomatal CO_2_ sensors remained to be identified (Hu et al. 2010).

### Direct modulation of SLAC1 anion channel by CO_2_/HCO_3_  ^−^

The *SLAC1* (*SLOW ANION CHANNEL-ASSOCIATED 1*) gene encodes an S-type anion channel and triggers stomatal closure by releasing anions from guard cells (Negi et al. 2008, Vahisalu et al. 2008). S-type anion channel currents are enhanced by intracellular bicarbonate in wild-type Arabidopsis guard cells (Hu et al. 2010, Xue et al. 2011). The CO_2_/HCO_3_^−^ signal is transduced to SLAC1 anion channel via protein kinases (Hashimoto et al. 2006, Xue et al. 2011, Hua et al. 2012, Hõrak et al. 2016). SLAC1 is activated by protein kinases (Geiger et al. 2009, Lee et al. 2009, Brandt et al. 2012). Electrophysiological reconstitution of the CO_2_ response can be examined by measuring SLAC1-mediated anion channel currents in *Xenopus laevis* oocytes. For example, the co-expression of SLAC1 with either the OST1 (OPEN STOMATA 1) protein kinase or CPK6 (Ca^2+^-dependent protein kinase 6) or CPK23 in oocytes was found to activate SLAC1-mediated anion channel currents (Geiger et al. 2009, Lee et al. 2009). Reconstitution of ABA activation of SLAC1 was demonstrated by co-expression of ABA receptors, protein kinases, and PP2Cs (Brandt et al. 2012). The following reconstitution experiments indicated the potential for the existence of multiple CO₂ signaling pathways. Co-expression of CAs, PIP2;1 aquaporin, protein kinases, and SLAC1 showed that SLAC1 channels are enhanced at elevated CO_2_/HCO_3_^−^, and this was observed independent of which protein kinase (OST or CPK6 or CPK23) was co-expressed in oocytes. SLAC1 activity is further directly enhanced by ≅20% by elevation in the cytosolic bicarbonate concentration (Zhang et al. 2018). The R256 residue in SLAC1 is important for this direct modulation by CO_2_/HCO_3_^−^ (Zhang et al. 2018). The structural resolution of the SLAC1 channel supports this finding, in which the R256 residue plays an important role in regulating the open probability of SLAC1 (Deng et al. 2021, Li et al. 2022). Moreover, the ten transmembrane-domain region of SLAC1 has been shown to function in CO_2_/HCO_3_^−^ sensing (Yamamoto et al. 2016). Gas exchange and patch clamp studies on transgenic plants with SLAC1-R256A demonstrated a partial impairment in CO_2_-regulated stomatal movement and HCO_3_^−^-activated S-type anion channel currents in guard cell membranes, while low CO_2_ elicited an intact stomatal opening response in SLAC1-R432A-expressing plants (Zhang et al. 2018). These observations suggested that the modulation of SLAC1 by CO_2_/HCO_3_^−^ is modulatory and requires protein kinases that regulate SLAC1 and that other primary CO_2_ sensors must exist that regulate protein kinases (Zhang et al. 2018).

## HT1-MPK4/12 Complex Is the Primary CO_2_/HCO_3_  ^−^ Sensor

### CO_2_ regulation of protein phosphorylation cascade

The Arabidopsis HT1 and CBC1 protein kinases were identified as negative regulators in CO_2_-induced stomatal closure. The strong recessive *ht1-2* allele shows no discernible low CO_2_-induced stomatal opening. Using recombinant proteins, Takahashi et al. found that the HT1 Raf-like kinase dramatically activates CBC1 protein kinase activity by phosphorylation at two amino acid residues inside or near the CBC1 kinase activation loop (Takahashi et al. 2022). The positive regulators MPK4/12 were found to inhibit HT1 activity (Hõrak et al. 2016, Jakobson et al. 2016, Takahashi et al. 2022). Addition of MPK4 or MPK12 revealed that adding NaHCO_3_ as a source of CO_2_/bicarbonate reduced HT1-induced CBC1 kinase activation *in vitro*. When HT1 and MPK4/12 were mixed without the addition of CBC1, HT1 kinase activity decreased in the presence of NaHCO_3_, while another pair of protein kinases alone (HT1 and CBC1) did not respond to NaHCO_3_. The results above indicate that the HT1 and MPK4/12 combination can sense CO_2_/HCO_3_^−^ (Takahashi et al. 2022).

### CO_2_/HCO_3_  ^−^ enhances HT1-MPK4/12 complex formation

HT1 and MPK4/12 were reported to interact with each other directly (Jakobson et al. 2016, Tõldsepp et al. 2018). *In vitro* pull-down assays and quantitative blinded BiFC experiments in plant cells revealed that the MPK4/12-HT1 interaction depends on the presence of CO_2_/HCO_3_^−^ (Takahashi et al. 2022). Several CO_2_-insensitive dominant mutations in the *HT1* gene, including *HT1-R102K* (Hashimoto-Sugimoto et al. 2016) and *HT1-A109V* (Hõrak et al. 2016), impaired the interaction of these HT1 variants with MPK4 and MPK12, which can explain their dominant high CO_2_-insensitive phenotype. Moreover, the HT1-R102K and HT1-A109V isoforms disrupted the NaHCO_3_-dependent CBC1 inhibition *in vitro* (Takahashi et al. 2022), suggesting that CO_2_/HCO_3_^−^-induced MPK4-HT1 complex formation is required for CO_2_-induced stomatal closure.

### MAP kinase activity is not required for CO_2_ sensing

**Figure 2:**
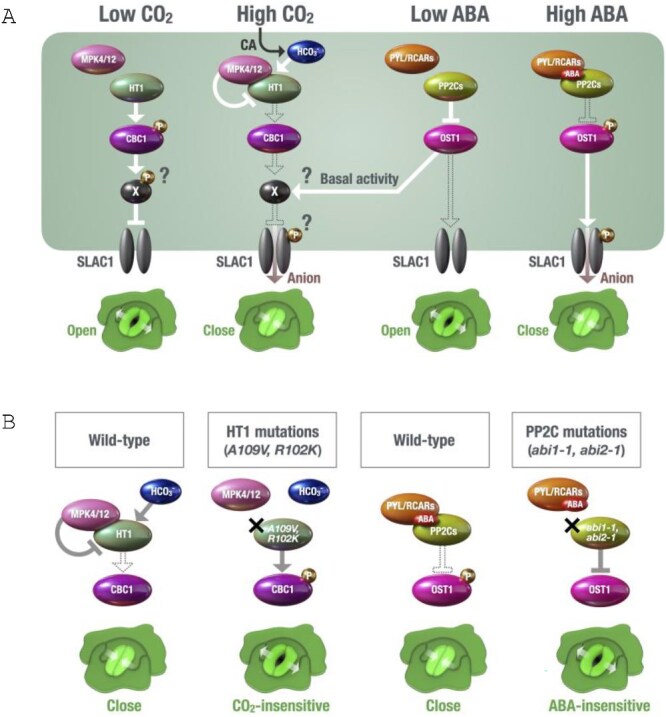
CO_2_ sensing and signal transduction in stomatal guard cells. (A) Schematic model of the early receptor/sensor signal transduction mechanisms of CO_2_ and ABA. Under low [CO_2_] conditions, Raf-like HT1 kinase phosphorylates and activates Raf-like CBC1 kinase, resulting in stomatal opening through unidentified CBC1 substrate(s) (“X”). In response to high [CO_2_], HCO_3_^−^ produced by β-CA induces binding of HT1 and MPK4/12 MAP kinases. MPK4/12 inhibits HT1 kinase activity and induces stomatal closure. Under non-drought-stress conditions (“Low ABA”), PP2Cs inhibit OST1/SnRK2 protein kinases by dephosphorylation. Although OST1/SnRK2s only have a basal kinase activity, this can aid in CO_2_-induced stomatal closure. In the presence of ABA, PYL/RCARs ABA receptors bind to and inhibit PP2Cs, which activates OST1/SnRK2 kinases and promotes stomatal closure. Both CO_2_ and ABA regulate S-type anion channels (SLAC1) as a common target at the PM. (B) The dominant mutations of HT1 (*A109V* and *R102K*) impair CO_2_ sensing by disrupting MPK4/12 binding. This can activate CBC1 and cause CO_2_-insensitive constitutive open stomata in these dominant mutant plants. These HT1 mutations may have a similar effect to the well-known ABA-insensitive PP2C mutations, *abi1-1* and *abi2-1*, which disrupt ABA-dependent binding to PYL/RCARs thereby impeding inhibition of their phosphatase activity, resulting in open stomata and strong ABA-insensitivity.

Surprisingly, the kinase-inactive MPK12-K76R sustained the ability to mediate the CO_2_/HCO_3_^−^-dependent CBC1 inhibition *in vitro* and rescued the CO_2_-less sensitive phenotype of the *mpk12* knockout mutant (Takahashi et al. 2022). These data suggest that CO_2_ sensing by HT1 and MPK4/12 does not seem to require the kinase activity of MPK4/12 (Takahashi et al. 2022). How the HT1 kinase is inhibited by MPK4/12 and elevated CO_2_/HCO_3_^−^ remains unknown. The complex formation with MPK4/12 may induce HT1 structural change or block the interaction with CBC1 or ATP. This molecular sensor mechanism could be similar to the ABA receptor mechanism, in which ABA inhibits PP2C phosphatase activity through the ABA receptor Pyrabactin Resistance 1-Like or Reulatory Components of ABA Receptor (PYL/RCARs) that, upon binding ABA, directly interacts with the PP2C catalytic site (Cutler et al. 2010). The findings that the dominant *HT1-R102K* and *HT1-A109V* point mutations inhibit CO_2_/HCO_3_^−^-dependent binding of these HT1 variants to MPK4/12 may indicate that these mutations could have a similar effect to the well-known ABA-insensitive PP2C mutations, *abi1-1* and *abi2-1*, which disrupt ABA-dependent binding to PYL/RCARs, thereby impeding inhibition of their phosphatase activity. The proposed CO_2_ sensing and signaling model overview is shown in Fig. 2.

### CO_2_ or HCO_3_^−^?

In the apoplastic space and cytoplasm, CO_2_ can dissolve in water and exists as CO_2_ and HCO_3_^−^ forms depending on the prevailing pH. The conversion between CO_2_ and HCO_3_^−^ carbon species is rapidly catalyzed by βCAs. HCO_3_^−^ can act as a signaling molecule in the stomatal CO_2_ response (Xue et al. 2011). The inhibition of HT1 kinase activity by MPK4 and NaHCO_3_ was observed in the reaction buffers adjusted to pH 7 or higher (Takahashi et al. 2022). Since HCO_3_^−^ is the predominant form in such pH conditions, HT1 and MPK4/12 may perceive mainly HCO_3_^−^ more so than CO_2_ as a ligand. However, further research will be required to address this hypothesis.

### Brachypodium BdMPK5 functions in CO_2_ sensing

Grass stomata have unique structures consisting of two guard cells, flanked by subsidiary cells (Raissig et al. 2016). The molecular mechanisms mediating stomatal CO_2_ signal transduction in grasses have remained largely unknown. A recent unbiased forward genetics infrared imaging CO_2_ response screen revealed that the Brachypodium *BdMPK5* gene, an orthologue of Arabidopsis *AtMPK4*, plays a central role in CO_2_-induced stomatal closure (Lopez et al. 2024). The *BdMPK5* mutant identified by a genetic thermography screen causes a single amino acid substitution of D90N in the BdMPK5 protein. *In vitro* phosphorylation assays showed that the BdMPK5-D90N protein could not inhibit HT1 kinase activity, whereas the wild-type BdMPK5 protein could inhibit HT1 upon NaHCO_3_ addition (Lopez et al. 2024). These results indicate that CO_2_ sensing by MAP kinase and HT1 may be conserved in monocots.

## Interactions between Stomatal CO_2_ Sensing and Environmental Conditions: Light, Temperature, ABA, and Atmospheric CO_2_

### Intercellular CO_2_ concentration levels are dynamically changed by stomatal conductance and photosynthesis.

Stomata provide an avenue for CO_2_ intake required for photosynthesis that largely occurs in leaf mesophyll cells. Stomatal conductance is positively correlated with CO_2_ assimilation over wide ranges of environmental conditions in C3 and C4 plants (Wong et al. 1979). For example, at higher light intensities and warming temperatures, photosynthesis increases, as do stomatal apertures (Raschke 1970, Pankasem et al. 2024). Stomatal conductance and net CO_2_ assimilation directly affect the intercellular CO_2_ concentration (*c_i_*). Direct measurements of leaf internal CO_2_ concentration using microelectrodes demonstrated a wide *c_i_* range with a rapid decrease of *c_i_* to the range of <~200 ppm in response to light exposure and increase in CO_2_ assimilation. Furthermore, *c_i_* increased rapidly up to ~three-fold within minutes after the onset of darkness due to a cessation of photosynthesis (Hanstein et al. 2001, Roelfsema et al. 2002). Such rapid and dynamic physiological ranges of leaf internal CO_2_ concentrations result in feedback causing stomatal opening and closing responses to achieve the optimal balance of photosynthetic capacity and WUE (Ball et al. 1987).

### Light and CO_2_ signaling in stomata

Red light and blue light induce stomatal opening. Red light is required at a much higher intensity than blue light to induce robust stomatal opening (Shimazaki et al. 2007). The blue light sensing and signaling mechanisms mediated by phototropins have been relatively well characterized and are reviewed elsewhere (Shimazaki et al. 2007). However, how the red light signal is perceived and transduced remains largely unknown and a matter of debate.

A body of evidence has shown that red light induces stomatal opening in Arabidopsis through its direct stimulation of leaf photosynthesis, primarily in mesophyll cells (Lawson et al. 2014). An increase in leaf photosynthesis under red light results in a reduction in *c_i_* and an increase in photoassimilates. A study showed that stomatal CO_2_ signaling mechanisms play a pivotal role in mediating red light-induced stomatal opening. It was found that low CO_2_ enhanced the stomatal blue light response (Assmann 1988). Hosotani et al. (2021) reported that a low CO_2_ background partially bypasses the strong red light background required for stimulating a robust blue light-induced stomatal opening. In addition, genetic studies performed by several independent groups showed that mutants lacking key CO_2_ signaling players showed impaired red light responses (Matrosova et al. 2015, Hiyama et al. 2017, Taylor et al. 2024). Note, however, there is a parallel, molecularly less well understood red light signal transduction pathway in guard cells that mediates stomatal opening, independent of Ci, which is observed in mesophyll-free leaf epidermal strips of *C. communis* (Schwartz and Zeiger 1984). Moreover, experiments in *Arabidopsis* did not exclude an additional *c_i_*-independent component in red light-induced stomatal opening (Matrosova et al. 2015).

Several studies performed leaf gas exchange experiments where the leaf internal CO_2_ concentration was controlled to a stable level and showed that stomata conductance increased in response to red light exposure (Messinger et al. 2006, Lawson et al. 2008). This suggests that an additional unknown red light sensing mechanism and signaling components can contribute to stomatal opening in response to red light and increased photosynthesis. Such low *c_i_*-independent mechanisms may, in part, explain the weak red light response in strong CO_2_ signaling mutants (Matrosova et al. 2015, Taylor et al. 2024). Whether there is a biochemical interaction of *c_i_*-independent red light and CO_2_ signaling mechanisms deserves thorough investigation in the future.

The leaves of *cbc1/cbc2* double mutant plants lacking Raf-like CBC1/CBC2 protein kinases, that function downstream of the HT1 Raf-like kinase, showed strongly impaired stomatal low CO_2_ and red light-induced stomatal opening (Hiyama et al. 2017). HT1 functions as a subunit of the CO_2_/bicarbonate sensor that directly phosphorylates and activates CBC1 (Takahashi et al. 2022). HT1 can be activated in response to low CO_2_/HCO_3_^−^ concentrations (Takahashi et al. 2022). Under red light, it is likely that the resulting low *c_i_* causes the level of HT1-mediated CBC1 phosphorylation to be increased in guard cells of intact leaves. CBC1/CBC2 has been genetically linked to a light-induced deactivation of S-type anion channels in guard cells (Hiyama et al. 2017).

In addition, when whole leaves were exposed to red light, the phosphorylation level at various phosphosites of the PM H^+^-ATPases in stomatal guard cells increased, resulting in stomatal opening (Ando and Kinoshita 2018, Fuji et al. 2024, Hayashi et al. 2024). The penultimate threonine residue of H^+^-ATPases is rapidly dephosphorylated upon transition to darkness, elevated CO_2_ or in the presence of NaHCO_3_ (Ando et al. 2022). Taken together, this suggests that CO_2_ and red light signal transduction mechanisms are likely to be integrated at the signaling events upstream of H^+^-ATPases, S-type anion channels, and other metabolic mechanisms, channels, and transporters in the guard cell signaling network, including the K^+^ uptake and K^+^ release channels in the PM of guard cells (Schroeder et al. 1987, Brearley et al. 1997).

### Warming temperatures and stomatal CO_2_ response

**Figure 3:**
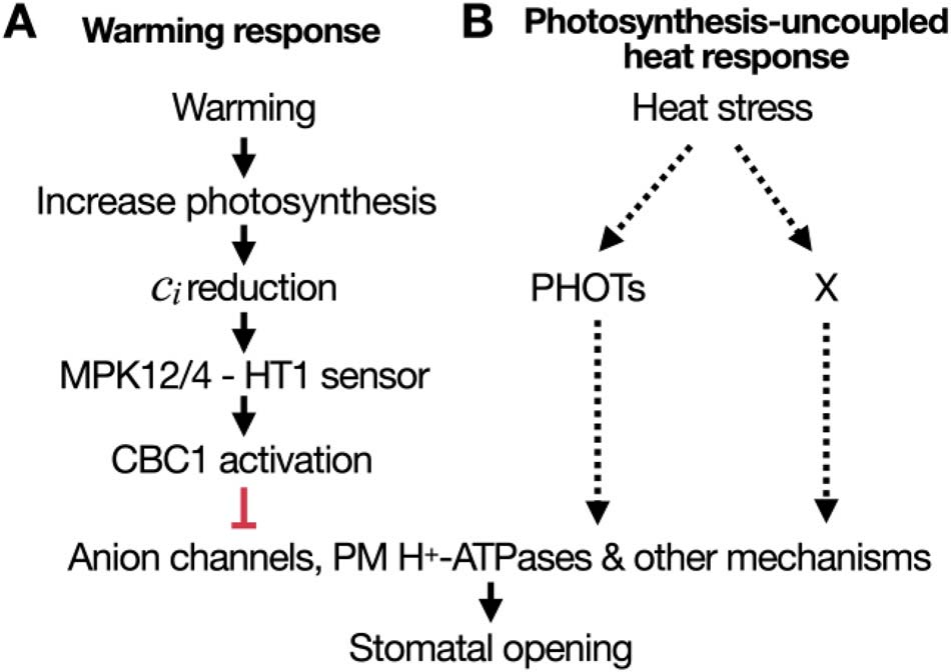
Distinct genetic and physiological mechanisms underlie stomatal opening to warming and heat stress. (A) Warming enhances photosynthesis, thus reducing intercellular CO_2_ concentrations (*c_i_*) and triggering stomatal opening via the CO_2_/bicarbonate MPK12/4-HT1 sensing complex and link to CBC1 activation at warming temperatures (Pankasem et al. 2024). (B) At higher temperatures, stomata further open when photosynthesis decreases, causing the decoupling of stomatal opening from photosynthesis. Kostaki et al. 2020 demonstrated a requirement of PHOT1/PHOT2 for the stomatal response to high temperature. The molecular mechanisms underlying warming and photosynthesis-uncoupled heat stress responses are likely upstream to the regulation of anion channels and the PM H^+^-ATPases.

Increasing temperatures cause stomatal opening (Darwin 1898). However, the genetic and molecular mechanisms have been difficult to investigate, as increasing temperatures also increase the vapor pressure difference (VPD_leaf_) between leaves and the atmosphere, which causes stomatal closing (Merilo et al. 2018). Experimental methods that hold VPD_leaf_ stable have enabled the first insights into this pathway. Recently, stomatal CO_2_ sensors and signaling mechanisms were found to mediate the stomatal response of intact leaves to warming temperatures. Stomatal opening mediated by a 10°C temperature increase from 18°C to 28°C was severely impaired in mutants disrupting the functions of CO_2_ sensors and early signaling components, including HT1, MPK12/MPK4, and CBC1/CBC2 (Pankasem et al. 2024). This temperature increase enhances CO_2_ assimilation by rapid photochemical and biochemical processes, which in turn causes a reduction in the leaf’s internal CO_2_ concentration (Raschke 1970, Farquhar and Wong 1984, Pankasem et al. 2024). Indeed, warming-induced stomatal opening was abolished in mutant leaves of the CO_2_ sensor component HT1. Combined genetic, physiological, and biochemical findings suggest that the warming-induced increase in leaf photosynthesis, followed by a reduction in *c_i_*, increases HT1 kinase activity (Takahashi et al. 2022, Pankasem et al. 2024) (Fig. 3).

Interestingly, stomatal conductance further increased when temperatures further increased to 34°C, but CO_2_ assimilation decreased (Urban et al. 2017, Diao et al. 2024, Pankasem et al. 2024). These findings show that additional photosynthesis-uncoupled mechanisms are triggered when plants are exposed to heat stress, most likely to facilitate evaporative cooling of leaves (Fig. 3). Several studies have recently proposed molecular mechanisms that could be linked to photosynthesis-uncoupled heat stress responses, including the stomatal blue light sensor phototropins (Kostaki et al. 2020). Furthermore, direct activation of PM H^+^-ATPases at 34°C by high-temperature-responsive MAP4K, TARGET OF TEMPERATURE 3 was proposed (Xu et al. 2024). Another hypothesis proposes that heat causes rapid degradation of ABA in guard cells (Mills et al. 2024). The mechanisms mediating heat stress-induced stomatal opening deserve further investigation, given the increasing occurrence of heat waves in many regions.

### CO_2_ and ABA response

The interaction between ABA and CO_2_ in stomatal closure has been debated for decades. Studies of the CO_2_ sensor mutants *ht1-2*, *ht1-A109V*, and *mpk12/mpk4GC* demonstrated that ABA-induced early guard cell signal transduction occurs in first order independently of the early CO_2_ signaling pathway (Hashimoto et al. 2006, Hõrak et al. 2016, Tõldsepp et al. 2018). This model is further supported by Boolean modeling (Gan et al. 2024) and physiological analyses showed that ABA induces stomatal closure at nominally zero CO_2_ (Karanam et al. 2021). In contrast, alterations in stomatal CO_2_ response kinetics observed in ABA signal transduction mutants point to a role for ABA in elevated CO_2_-induced stomatal closure (Xue et al. 2011, Chater et al. 2015, Hsu et al. 2018, Zhang et al. 2020, Seller and Schroeder 2023). A study suggested high CO_2_ signal transduction occurs via the upregulation of ABA receptor transcripts and ABA signal transduction (Dittrich et al. 2019). However, high CO_2_-induced stomatal closing is completed within minutes, whereas transcript up-regulation and protein synthesis usually require more time. Stomatal CO_2_ response phenotypes in the ABA receptor mutants reported by Dittrich et al. (2019) could not be confirmed (Zhang et al. 2020). In addition, elevated CO_2_ was found not to activate ABA signal transduction based on the following findings: CO_2_ elevation did not rapidly increase ABA concentrations in guard cells nor ABA-induced gene expression in guard cells nor activate OST/SnRK2s protein kinases in guard cells (Hsu et al. 2018, Zhang et al. 2020). Interestingly, though, plants maintain higher basal ABA levels and basal OST1/SnRK2s protein kinase activities in guard cells compared to other leaf cells under non-stressed conditions (Lahr and Raschke 1988, Hsu et al. 2018, Zhang et al. 2020). The current model suggests that basal ABA signaling amplifies and facilitates CO_2_-induced stomatal closure, with ABA and CO_2_ signal transduction converging downstream of OST1/SnRK2s protein kinases in guard cells (Hsu et al. 2018, Zhang et al. 2020).

ABA induces massive chromatin remodeling in guard cells, and ABA-induced accessible regions in guard cells are substantially more abundant and significantly affect different accessible chromatin regions than those regulated by low or high CO_2_ conditions, demonstrating that ABA and CO_2_ cause highly distinct transcriptional programs in guard cells (Seller and Schroeder 2023). The above findings correlate with the role of ABA signaling in generating a ‘memory’ effect in guard cells during drought. ABA signaling is proposed to be required for building transcriptional memory in guard cells to prevent immediate reopening after re-watering plants and ensures a faster response to subsequent repeated dehydration stress (Virlouvet and Fromm 2015, Seller and Schroeder 2023). Typically, plants cannot reopen their stomata until several days after re-watering has occurred. In contrast to drought, stomata can open and close rapidly and reversibly in response to fluctuations in intracellular CO_2_ concentrations driven by the balance of photosynthetic activity and dark respiration throughout the day. Interestingly, several accessible chromatin regions upstream of some positive regulators of low CO_2_-induced stomatal opening are specifically repressed by ABA (Seller and Schroeder 2023), indicating a cross-talk mechanism between the ABA and CO_2_ response at the level of chromatin remodeling, potentially enabling drought-induced stomatal closing to dominate over low CO_2_-induced stomatal re-opening (Seller and Schroeder 2023). These findings further indicate that basal ABA can modulate the stomatal CO_2_ response by fine-tuning chromatin accessibility and the expression level of CO_2_ signaling genes.

### Stomatal development regulation by CO_2_

Elevated CO_2_ concentrations cause a reduction in stomatal density (Woodward 1987). Mutant leaves lacking the *HIGH CARBON DIOXIDE* encoding an enzyme that produces very long-chain fatty acids and wax in guard cells show an increased stomatal index under a high CO_2_ concentration in contrast to the lower stomatal index of WT plants (Gray et al. 2000). Such an inverse phenotype was also found in mutant leaves of the negative regulators of stomatal development *epf2* and *erecta*, disrupting genes encoding pro-peptide and a peptide ligand binding leucine-rich receptor protein kinase, respectively (Engineer et al. 2014). Interestingly, *ca1/ca4* double mutant leaves lacking CA isoforms that show slowed stomatal conductance responses to CO_2_ concentration changes (Hu et al. 2010, Kolbe et al. 2018) show increased stomatal density and index in Arabidopsis (Engineer et al. 2014).

Although *ca1/ca4* showed a stomatal development phenotype (Hu et al. 2010, Engineer et al. 2014), other strong CO_2_ signaling mutants, including *ht1* mutants and *mpk12/mpk4* mutants, do not show a clear difference in stomatal density (Hashimoto et al. 2006, Engineer et al. 2014, Hõrak et al. 2016, Tõldsepp et al. 2018). Therefore, the HT1-MPK12/MPK4 mediated CO_2_/bicarbonate sensing mechanism does not appear to function upstream of CO_2_-modulation of stomatal development. The physiological mechanisms by which CAs affect stomatal development require further analysis.

## Dual-Role Kinase MPK4: Plant Immune Response and CO_2_ Sensing

### MPK4 is a central MAP kinase in the pathogen response pathway

**Figure 4:**
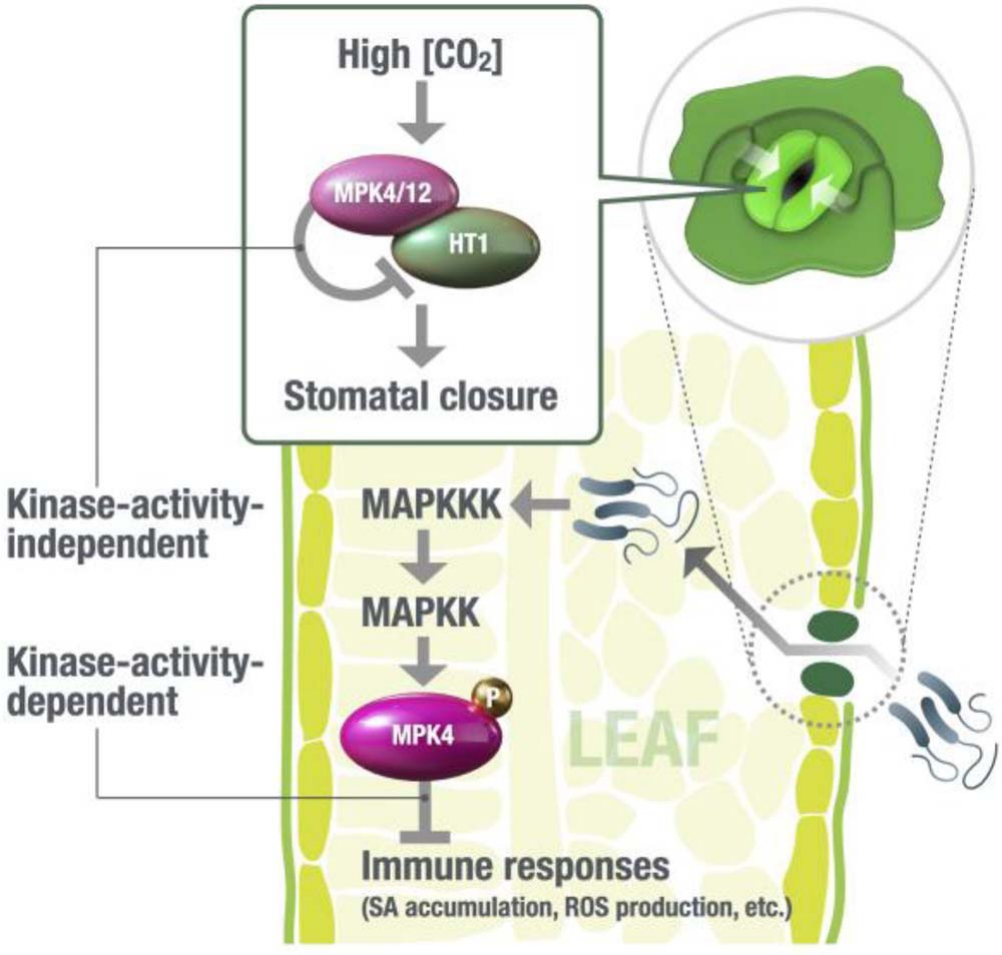
MPK4 functions in stomatal CO_2_ sensing and plant immune response, but the mechanisms are different. In guard cells, MPK4 and MPK12 inhibit HT1 kinase activity by direct binding in response to high CO_2_ concentrations. MAP kinase activity is not required for the stomatal CO_2_ response. In leaf cells, MPK4 mediates immune responses. The MPK4 function in the immune response depends on its protein kinase activity regulated by the upstream MAPKK and MAPKKK. MPK12 is unlikely to have an important role in immune signal transduction. Since HT1 expression in other leaf cell types other than guard cells is weak to absent, HT1 may not function in the MPK4-dependent immune response pathway. Further research is needed to address whether there is any interaction between stomatal CO_2_ sensing and immune responses in leaves.

MPK4 plays important roles in many plant immune responses against pathogens. Arabidopsis *mpk4* loss-of-function mutants show an extreme dwarf phenotype, salicylic acid accumulation, and enhanced immune-related gene expression, suggesting that MPK4 negatively regulates the immune response in Arabidopsis (Petersen et al. 2000). MPK4 is activated by a classical upstream MAP kinase cascade consisting of MAP3K (MEKK1) and MAP2K (MKK1/2) in immune signal transduction (Fig. 4) (Berriri et al. 2012). This suggests that MPK4 kinase activity is required for the plant pathogen response.

The extreme dwarf phenotype of *MPK4* is not complemented by *MPK12* expression under the control of the *MPK4* promoter (Tõldsepp et al. 2018). MPK4 and MPK12 share 81% amino acid identity (BLASTP alignment), and both MPKs function in CO_2_ sensing in Arabidopsis, but their immune response functions differ. The observation that recombinant MPK12 protein has a weaker kinase activity than MPK4 (Tõldsepp et al. 2018) is consistent with the inability of *MPK12* to complement the *mpk4* mutant’s dwarf phenotype. To investigate how MPK4 and MPK12 proteins function differently may provide insight into how the immune response and the stomatal CO_2_ response are specifically transduced via the same signaling component.

### MPK4 homologs in *P. patens*

The moss *P. patens* has two homologs of Arabidopsis *AtMPK4*, named *PpMPK4a* and *PpMPK4b*, and it has been shown that *PpMPK4a* is required for immune responses (Bressendorff et al. 2016). This suggests that the involvement of *MPK4* in plant immune responses seems to be an ancient function.

A previous study reported that *P. patens* closes stomata in response to high CO_2_ concentrations (Chater et al. 2011). Furthermore, chloroplast orientation movement is regulated in response to CO_2_ in the moss (Sugiyama and Terashima 2023). These findings suggest that *P. patens* has CO_2_ sensing mechanism(s). However, whether moss stomata can respond to CO_2_ is controversial (Brodribb and McAdam 2011). Another study from a different group reported that stomatal conductance does not respond to CO_2_ concentration changes in several moss species (Kubásek et al. 2021). It is unknown whether the moss MPK4s function in CO_2_ sensing. Investigating the function of PpMPK4a and PpMPK4b proteins in CO_2_ sensing and the evolutionary history of HT1 may provide insight into the origin of the plant stomatal CO_2_ response. However, recent phylogenetic analyses have shown that HT1 originated from angiosperms (Ivsic et al. 2025).

### Gene duplication produces MPK4 family members with different roles

It has been suggested that the Arabidopsis *MPK12* gene arose from an ancestral *MPK4* gene in the *Brassicaceae* (Tõldsepp et al. 2018). The Arabidopsis genome includes *MPK11*, the closest homologous gene to *MPK4* (89% amino acid identity in BLASTP alignment), in subgroup B of the MAP kinase gene family. MPK4 plays an important role in cell plate formation by interacting with microtubules during cell division, and MPK11 seems to play a partially redundant role in this process (Kosetsu et al. 2010). Surprisingly, MPK11 does not seem to function in the stomatal CO_2_ response (Tõldsepp et al. 2018). *P. patens MPK4b* may have a more crucial or broader function than *PpMPK4a* since the authors could not isolate a knockout mutant in the moss *PpMPK4b* gene (Bressendorff et al. 2016). These imply that the *MPK4* genes among plant species might have evolutionarily developed diversified functions by gene duplication.

### Stomatal CO_2_ sensing and plant immune response pathways

One of the open questions is why MPK4 plays important roles in two different environmental responses: immune response and stomatal CO_2_ response. Stomata are important for the immune response since stomatal pores provide a pathway for pathogen entry into plant leaves. A previous study revealed that plants close stomata in response to pathogen elicitors, referred to as stomatal immunity (Melotto et al. 2006). On the other hand, pathogens secrete effectors that have been suggested to promote stomatal opening to facilitate invasion into the plant body. A recent study further showed that bacterial effectors also promote stomatal closure via ABA accumulation to induce an aqueous space in the apoplast (Roussin-Leveillee et al. 2022). Stomatal signaling may have evolved through interactions between plants and pathogens. It is unclear whether MPK4 is involved in the pathogen-induced responses in stomata. Recently, the *cbc1 cbc2* mutant was reported to show resistance to pathogens (Goncalves Dias et al. 2024), which may be a direct pleiotropic effect of the reduced stomatal conductance of this double mutant that restricts the entry of the pathogens (Hiyama et al. 2017). More genetic and physiological evidence will be needed to understand the possible crosstalk of these two responses.

## Future Research

The discovery of CO_2_/HCO_3_^−^-dependent complex formation of HT1 and MPK4/12 has revealed the nature of plant stomatal CO_2_ sensing. HT1-induced CBC1 activation and CO_2_-induced inhibition of HT1 kinase activity by binding MPK4/12 have provided insight into the primary CO_2_ signaling core. These findings have also highlighted some questions to be addressed in the future. Solving the structure of the HT1-MPK4/12 protein complex will be necessary. The precise structure of the HT1-MPK4/12 complex would solve these important questions: (i) whether and, if so, how CO_2_ and/or HCO_3_^−^ bind to this protein complex? (ii) How does CO_2_/HCO_3_^−^ enhance HT1-MPK4/12 complex formation? (iii) How do MPK4/12 and HT1 specifically bind to each other? And (iv) How does MPK4/12 inhibit HT1 kinase activity in the presence of CO_2_/HCO_3_^−^ without MAP kinase activity (Takahashi et al. 2022)? Additionally, the direct downstream substrate(s) of CBC1/2 protein kinases and protein phosphatases that have been proposed to be required to dephosphorylate CBC1 and possibly HT1 (Takahashi et al. 2022) remain unknown.

Stomatal regulation of gas exchange is critical for plant WUE. Studies in crops have provided evidence that WUE can be improved at elevated CO_2_ concentrations (Conley et al. 2001, Al-Salman et al. 2023, Sobuj et al. 2024, Radolinski et al. 2025). This effect is considered due to decreased stomatal conductance and transpirational water loss and potential increased carbon assimilation under high CO_2_ concentration conditions. Note that WUE depends on conditions and plant species, given the impacts of heat stress and vapor pressure deficit on stomatal conductance and water use (Gray et al. 2016). Genetic evidence has documented that the stomatal CO_2_ response is critical for improving instantaneous WUE (Hu et al. 2010, Des Marais et al. 2014, Xiao et al. 2024). The HT1 ortholog in rice likely functions in the stomatal CO_2_ signaling pathway (Xiao et al. 2024). To investigate whether the HT1-MPK4/12 CO_2_/HCO_3_^−^ sensor is conserved in other crops will be needed. The effect of high CO_2_ concentrations on WUE could be affected by temperature and water stress (Gray et al. 2016, Hatfield and Dold 2019, Li et al. 2023, Pankasem et al. 2024, Sobuj et al. 2024). Further research on the molecular interactions between these signaling pathways would advance our knowledge.

Atmospheric CO_2_ concentrations have fluctuated since stomata emerged 400 million years ago. It remains unclear when land plants acquired a stomatal movement CO_2_ response. The evolutionary aspect of the HT1-MPK4/12 module and signaling factors such as CBC1 would be interesting subjects for additional analyses. Understanding how plants have responded to and acclimated to atmospheric CO_2_ concentrations may provide a clue for future efficient plant breeding and engineering to improve crop yields and WUE of crops and trees in view of increasing CO_2_ concentrations and weather extremes.

## Data Availability

Data available on request.
